# 

**Published:** 1826-07

**Authors:** 


### Contents

OF

No. 9. Vol. V. (NEW SERIES) for JULY, 1826.

[*No. 25, ANALYTICAL SERIES.*]

Art. I.

FEVER—ITS SEAT AND NATURE.

1. Conversations on Physiological Medicine, by a Disciple of Broussais. .. .. .. .. .. .. .. .. ..

2. An Enquiry into the Seat and Nature of Fever. By Henry Clutterbuck, M.D. (Second Edition.).. .. .. ..

3. Examen des Doctrines Medicales, &c. Par M. Broussais, M.D.. .. .. .. .. .. .. .. .. .. .. } 1

II.

Transactions of the Medical and Physical Society of Calcutta, vol. 1.. .. .. .. .. .. .. .. .. .. .. .. 36

1. Fatal bite of a Venomous Snake .. .. .. .. .. .. 37

2. Wound of the Stomach.. .. .. .. .. .. .. .. 38

3. Oil in the Blood .. .. .. .. .. .. .. .. .. 39

4. On Delirium Tremens .. .. .. .. .. .. .. .. 40

5. On Adherent Calculus .. .. .. .. .. .. .. .. 41

6. On a Peculiar Eruptive Disease .. .. .. .. .. .. 42

7. On Dracunculus .. .. .. .. .. .. .. .. .. 42

8. On Aneurism of the Aorta .. .. .. .. .. .. .. 43

III.

Dr. Granville's Essay on Egyptian Mummies.. .. .. .. .. 46

IV.

THE MEDICINAL LEECH.

1. Dr. Johnson's Two Essays on the Leech.. .. .. .. ..
2. Article, "Sangsue." Par M. Merat .. .. .. .. ..
3. A Treatise on Sanguisuction. By Dr. Price.. .. .. } 52

V.

Dr. Scudamore on the Parisian Practice .. .. .. .. .. .. 73

VI.

M. Beaupré on the Historical and Medical Effects of Cold in the Russian Campaign .. .. .. .. .. .. .. .. .. 77

VII.

An Enquiry concerning that Disturbed State of the Vital Functions usually denominated Constitutional Irritation. By Benjamin Travers, Esq. (First Article.) .. .. .. .. 95

VIII.

Novum Organum Medicorum; or, New Medical Logic. By Professor Lanza, of Naples. (First Article.).. .. .. 182

IX.

Practical Observations on the Convulsions of Infants. By John North, Surgeon and Accoucheur .. .. .. .. .. .. 132

X.

### Quarterly Periscope

OF

### PRACTICAL MEDICINE;

BEING

*THE SPIRIT OF THE MEDICAL JOURNALS,*

FOREIGN AND DOMESTIC.

I. PHYSIOLOGY.

Dr. Troillet on the Cicatrization of Intestinal Ulcers .. .. .. 192
Dothinenteritis—Dr. Bretonneau's New Theory of Fever .. .. 199
M. Andral on the Cicatrization of Tuberculous Excavations in the Lungs.. .. .. .. .. .. .. .. .. .. .. 214
Dr. Dill on Cutaneous Absorption .. .. .. .. .. .. .. 240
Mr. Abraham on the Influence of Civilization .. .. .. .. 265

II. PATHOLOGY.

Dr. Malden's Fatal Cases of Bronchocele.. .. .. .. .. .. 189
Curious and Fatal Case of Mania, by Dr. Brierre.. .. .. .. 203
M. Bricheteau on the Pathology of Asthma.. .. .. .. .. 217
Prize-Essays on Insanity .. .. .. .. .. .. .. .. .. 226
M. Louis' curious Case of Sub-acute Metritis combined with Phlebitis. .. .. .. .. .. .. .. .. .. .. 242
Case of Melanosis of the Stomach, by M. Andral, Jun. .. .. 244
On the Rupture of Aneurisms in the Brain.. .. .. .. .. 249
Mr. Jowett's Case of Bronchitis succeeded by Pneumonia and Pericarditis .. .. .. .. .. .. .. .. .. .. .. 255
Singular Instance of Vomiting of Fat and Blood.. .. .. .. 247
Mr. Montgomery on the Contagious Nature of Cholera Morbus.. 259
A Case of Stricture and Perforation of the Colon .. .. .. 275
Sudden Death from Inflammation of the Peritoneum .. .. .. 276

III. SURGERY.

M. Gondret on a New Mode of curing Cataract.. .. .. .. 177

Operation for an Imperforate Anus. .. .. .. .. .. .. 187
Improved Operation for Staphyloraphy. .. .. .. .. .. 188
Mr. Mitchell's Case of Wound in the Lungs .. .. .. .. 204
Baron Larrey on Actual Cautery in Traumatic Erysipelas. . .. 205
Sea-water in Ophthalmia .. .. .. .. .. .. .. .. 222
Case of Extirpation of the Parotid Gland, by Dr. Prieger. . .. 223
Mr. George Bell on Excision in Cases of Wounded Nerves. . .. 245
Case of Partial Fracture of the Os Femoris, by Mr. B. Bell. . .. 249
M. Richerand's Progress of Surgery—illiberality of the French Critics .. .. .. .. .. .. .. .. .. .. .. 253
Fatal Case of Popliteal Aneurism, by Mr. Allan. . .. .. .. 257
Mr. Higginbottom on Lunar Caustic in Inflammation. . .. .. 260
Mr. Richmond on Cataract in India .. .. .. .. .. .. .. 263
A Case of Empyema successfully Treated by Paracentesis Thoracis. By Thomas Jowett, Esq. .. .. .. .. .. .. .. 267

IV. THERAPŒIA.

Dr. Carter's Hospital Reports .. .. .. .. .. .. .. .. 195
M. Bourgeois' Case of Scirrhus of the Stomach. . .. .. .. 206
Parallel between Cerebral Fever and Worm Fever .. .. .. 219
Observations on Bleeding .. .. .. .. .. .. .. .. .. 232
Dr. Maxwell's Remarks on Sea-sickness .. .. .. .. .. .. 251
Case illustrative of the Efficacy of the Acetate of Morphine applied externally .. .. .. .. .. .. .. .. .. .. 252
Messrs. Bland and Comte on the powers of Digitalis in Palpitatio Cordis .. .. .. .. .. .. .. .. .. .. .. .. 277

V. MIDWIFERY.

Mr. Jackson's Case of Difficult Parturition. . .. .. .. .. .. 198
Dr. Collin's Case of Eleven Month's Pregnancy. . .. .. .. .. 212
Case of Rupture of the Linea Alba in Pregnancy. . .. .. .. 242
Case of Rupture of the Uterus in the Third Month of Pregnancy 274
Medical Evidence on the Duration of Human Pregnancy. . .. 170
Extra-uterine Fœtus, successfully removed. . .. .. .. .. 275

VI. MEDICAL JURISPRUDENCE AND HYGIENE.

The Letter of Medico-Chirurgus on "a New Set of Physicians." 209
The Chevalier de Butel on Plague in the Eastern Countries. .. 233
Dr. Torrie on Poisoning by Cantharides .. .. .. .. .. .. 240
Dr. Bardsley's Case of Hydrophobia Simulata, with Remarks . . 261
M. Georgini on remedying the Insalubrity of Marshes .. .. 262

XI.

ANALECTA MINORA; OR MINOR PERISCOPE.

1. Sulphate of Cinchonine. . .. .. .. .. .. .. .. 283

2. Rupture of the Spleen .. .. .. .. .. .. .. .. 283

3. Hydriodate of Potash .. .. .. .. .. .. .. .. 284

4. Cauterization of Variolous Pustules .. .. .. .. .. .. 284
5. Successful Case of Cæsarian Section .. .. .. .. .. .. 284
6. Case of Tuberculated Peritoneum.. .. .. .. .. .. 284
7. On Bark as an Antidote to Tartar-emetic .. .. .. .. .. 285
8. Secale Cornutum .. .. .. .. .. .. .. .. .. 285
9. Novel Mode of Treating Retention of Urine.. .. .. .. 285
10. A Case of Local Spontaneous Combustion.. .. .. .. 286
11. M. Biel on the Antiphlogistic Treatment of Cancer .. .. 286
12. A Case of Emphysema Post Partum .. .. .. .. .. .. 287
13. Remarkable Instance of Diseased Bladder .. .. .. .. .. 287
14. Dr. Ravin on the Papillæ of the Tongue .. .. .. .. .. 288
15. Removal of the Alveolar Processes of both Jaws.. .. .. 288
16. Dr. Pallas on the Management of Leeches .. .. .. .. 289
17. Ictus Solaris—Rheumatism—Extensive Thoracic Disease.. 290
18. The Black Drop, an Old Preparation.. .. .. .. .. 291
19. On Antimoniated Ointment .. .. .. .. .. .. .. .. 291
20. Perforated Intestine—Artificial Anus cured.. .. .. .. 292
21. Sudden Congestion and Death .. .. .. .. .. .. .. 292
22. Curious Case of Strictured Œsophagus.. .. .. .. .. 293
23. Melanic Cancer of the Cheek.. .. .. .. .. .. .. .. 294
24. Cotemporaneous Rubeola and Variola.. .. .. .. .. 294
25. Doses of Calomel in Days of Yore.. .. .. .. .. .. 295
26. Neuralgia of the Scalp cured by an Operation .. .. .. 295
27. M. Lisfranc on Diseased Joints .. .. .. .. .. .. .. 296
28. On the Means of Ascertaining Infanticide.. .. .. .. 297
29. M. Thomas on Colica Pictonum.. .. .. .. .. .. .. 298
30. Curious Application of the Chloruret of Lime .. .. .. 298

XII.

Bibliographical Record.. .. .. .. .. .. .. .. .. .. .. 299

XIII.

MEDICAL MISCELLANY.

English Medical Etymologies .. .. .. .. .. .. .. .. 304

Viper-bite, Successful Application of the Cupping-glass .. .. 306

Angina Pectoris .. .. .. .. .. .. .. .. .. .. .. 307

Transfusion .. .. .. .. .. .. .. .. .. .. .. .. 307

XIV.

INTELLIGENCE, &c.

Queries and Speculations .. .. .. .. .. .. .. .. .. 308

Expeditious Warm Bathing.. .. .. .. .. .. .. .. .. 309

Mr. Kennedy's Improved Cupping Apparatus .. .. .. .. 309

Hospital Reports.. .. .. .. .. .. .. .. .. .. .. 309

List of Subscribers .. .. .. .. .. .. .. .. .. .. .. 312

